# Effects of Marine Phospholipids Extract on the Lipid Levels of Metastatic and Nonmetastatic Prostate Cancer Patients

**DOI:** 10.1155/2014/249204

**Published:** 2014-08-13

**Authors:** Daniela Küllenberg de Gaudry, Lenka A. Taylor, Jessica Kluth, Tobias Hübschle, Jonas Fritzsche, Bernd Hildenbrand, Lars Pletschen, Karin Schilli, Arwen Hodina, Lee S. Griffith, Jürgen Breul, Clemens Unger, Ulrich Massing

**Affiliations:** ^1^Department of Lipids & Liposomes, Tumor Biology Center, Breisacher Straße 117, 79106 Freiburg, Germany; ^2^Hermann Staudinger Graduate School, the University of Freiburg, Hebelstraße 27, 79104 Freiburg, Germany; ^3^Pharmacy Department, University Hospital Heidelberg, Im Neuenheimer Feld 670, 69126 Heidelberg, Germany; ^4^Loretto Hospital, Mercystraße 6-14, 79100 Freiburg, Germany; ^5^Urological practice U3, Bertoldstraße 45, 79098 Freiburg, Germany; ^6^PgXpertise, Bahnhofstraße 8, 79288 Gottenheim, Germany; ^7^Center for Cancer Medicine, Breisacher Straße 84 B, 79110 Freiburg, Germany; ^8^Department of Clinical Research, Tumor Biology Center, Breisacher Straße 117, 79106 Freiburg, Germany

## Abstract

High intake of omega-3 fatty acids (n-3 FAs) from fish has shown to reduce metastatic progression of prostate cancer. This clinical trial investigated the influence of high n-3 FA intake (marine phospholipids, MPL) on the FA composition of blood lipids, lysophosphatidylcholine (LPC), and on lipoproteins in prostate cancer patients and elderly men without prostate cancer. MPL supplementation resulted in a significant increase of n-3 FAs (eicosapentaenoic and docosahexaenoic acid) in blood lipids, while arachidonic acid (n-6 FA) decreased significantly. Low density lipoprotein (LDL) and high density lipoprotein (HDL) increased significantly, but the LDL increase was observed only in subjects with an inactive tumour. Similarly, LPC plasma concentration increased significantly only in patients without tumour. The missing increase of LDL and LPC after MPL supplementation in patients with actively growing (metastasizing) prostate cancer suggests that tumour cells have an elevated demand for LDL and LPC. Due to the MPL-induced increase of n-3 FAs in these blood lipids, it can be assumed that especially actively growing and metastasizing prostate cancer cells are provided with elevated amounts of these antimetastatic n-3 FAs. A hypothetic model explaining the lower incidence of metastatic progression in prostate cancer patients with high fish consumption is presented.

## 1. Introduction

Prostate cancer (PCa) is one of the most common cancers in men over the world [1]. Dietary factors have been found to play an important role during PCa and special attention has been given to dietary fat. An important study by Augustsson et al. showed that a high fish intake (>3 times per week) resulted in approximately 40% reduction of aggressive, metastatic PCa but did not influence the overall PCa incidence [2]. Similar effects were confirmed by a meta-analysis, in which a high fish consumption was demonstrated to lower the incidence of metastatic PCa by 44% and its mortality by 63% [3]. This effect has been shown to be mainly attributable to the n-3 fatty acids (FAs) found in fish [4].

The mechanisms by which n-3 FAs could have the potential to impede the formation of metastases are probably based on n-3 FAs as precursors of eicosanoids, which are less prometastatic and less inflammatory than eicosanoids derived from n-6 FAs [5]. Eicosanoids are lipid mediators, tissue hormones with autocrine or paracrine activity, which are involved in many physiological processes such as platelet function, immune response, and pain modulation. Dysregulation of eicosanoid biosynthesis has been linked to inflammation, infertility, allergy, degenerative diseases, atherosclerosis, ischemia, metabolic syndrome, and, most importantly, to cancer [6]. For eicosanoid biosynthesis, FAs have to be liberated from membrane phospholipids by lipolytic enzymes, which are predominantly phospholipases A2. Thereafter cyclooxygenases (COX) or lipooxygenases (LOX), which are the first step enzymes in the transformation of n-3 or n-6 FAs, convert the previously liberated FAs into different eicosanoids. An important example is the biosynthesis of prostaglandins E (PGE) by COX. PGE_2_, which is derived from arachidonic acid (AA), is highly active in promoting metastatic spread and aggressive tumour growth while PGE_3_, derived from the n-3 FA EPA, is less active [5, 7–9]. This, and the fact that the conversion of EPA is faster than the conversion of AA by the same enzyme (COX), n-3 FAs have the potential to act antimetastatic [5]. Thus, in tumour cells which often have an increased eicosanoid biosynthesis, the n-3/n-6 FAs ratio in the cellular membranes could determine the aggressiveness of the tumour cells.

However, to change the membrane n-3/n-6 FA ratio, the cells have to be supplied with the respective essential FAs. In blood, FAs are predominantly transported by lipoproteins. From there, FAs can be supplied to the cells, either as lysophosphatidylcholine (LPC) after the action of LCAT (lecithin-cholesterol-acyltransferase) [10], as free FAs after their cleavage from other lipoproteins like LDL, VLDL, or IDL by lipases, or taken up with the whole LDL lipoprotein via its receptor [11].

As discussed above, the incidence of metastatic PCa has shown to be reduced by about 40–50% if patients ingested high amounts of n-3 FAs [2]. During this clinical trial we investigated the interplay between metastatic growth of PCa and the effects of n-3 FA uptake on (i) blood FA composition (n-3/n-6 FA ratio), (ii) lipoprotein-, and (iii) LPC levels in patients with PCa as well as in elderly men without PCa (controls). The clinical trial was performed in the south western region of Germany, where fish consumption is usually low due to its scarcity in that area. Therefore, the study population consisted of low fish consumers. To establish FA-, lipoprotein, and LPC levels representative for high fish consumption, all subjects were supplemented with n-3 FAs given as marine phospholipids (MPL) for 3 months.

MPL is an n-3 FA-rich extract from salmon roe containing high amounts of phospholipids (PLs) containing the n-3 FAs eicosapentaenoic acid (EPA) and docosahexaenoic acid (DHA). Its composition (approximately 1/3 of the n-3 FAs are bound to PLs and 2/3 are bound to triglycerides) resembles the FA composition of fish better than other n-3 FA supplements like fish oil, which contains no PLs [12]. It has been shown that the uptake and utilization of n-3 FAs bound to PLs is more efficient than those bound to triglycerides (TGs) [13–15], but the metabolism of dietary ingested PLs has not been thoroughly investigated and many mechanisms remain to be clarified. However, it has been shown that, unlike TGs, they are almost completely absorbed as free fatty acids (FFAs) and LPC in the intestine. Following absorption, they are reesterified into PLs and incorporated mainly into chylomicrons to enter the bloodstream. Also, about 20% of the ingested PLs are absorbed passively and incorporated directly into high density lipoproteins (HDL) [16]. Additionally, other investigations with the same MPL formulation have found positive effects, for example, in the study of Taylor et al. patients with tumour associated weight loss (cachexia) achieved weight stabilization after MPL supplementation. These effects seem to be larger when compared to formulations of n-3 FA bound only to TGs. Therefore, supplementation with MPL is expected to be more effective in a relatively lower dose than fish oil or ethyl ester formulations regarding eicosanoid synthesis and its implications. In a previous study, a daily dose of 1.5 g MPL was shown to be enough to considerably change the n-3/n-6 FA ratio [17], while a study supplementing fish oil had similar effects, but with double the dosage (3 g n-3 FAs daily) [9].

## 2. Materials and Methods

### 2.1. Study Characteristics

The “Prostagen study” (registration ID: DRKS00000319 and UTN: U1111-1113-4482) was a cohort clinical trial in which patients with prostate cancer (PCa) and subjects without cancer (control group) were supplemented with marine phospholipids extract (MPLs). The clinical trial was designed based on the primary outcome of the study, which is not going to be addressed in this paper. Here, we describe the results of the trial in relation to the effects of MPL supplementation on the lipid and FA levels on the study population (secondary outcomes).

### 2.2. Study Population

The study population consisted of patients with prostate cancer (PCa) and subjects without PCa. All participants signed an informed written consent before study enrolment. The study protocol was approved by the “Ethics Committee of Freiburg University.” Patients were identified and recruited in Freiburg in three different centres, at the department of medical oncology at the “Tumor Biology Center,” the urological ward in the “Loretto Hospital” and the “Urological Practice U3” for a period of 23 months between December 2009 and October 2011.

PCa patients were included with various cancer stages, receiving different therapies, for example, surgery, radiation, hormone therapy, chemotherapy, or active surveillance. A total sample size of 150 patients (50 patients without PCa, 50 patients with PCa and Gleason score ≤ 7a, and 50 patients with PCa and Gleason score ≥ 7b) was calculated according to the primary endpoint of the study.

Inclusion criteria for PCa patients were male subjects older than 18 years with histologically proven PCa and who signed a written informed consent. Inclusion criterions for subjects without PCa were male subjects without PCa or any other tumour, aged 70 years or older (According to the American Cancer Society, the median age of PCa diagnosis is 66 years [18]. In order to comply with the certainty (needed for analyzing the primary endpoint of the study) that patients without PCa would not become PCa in their remaining lifetime, patients without PCa were to be 70 years or older. This brings selection bias into the study. However, this risk was taken, since it does not affect the primary endpoint of the study.). PCa diagnosis was defined as being excluded by the following criteria: (1) negative results from a 12x prostate punch biopsy and PSA values below 10 ng/mL; (2) individuals with benign prostatic hyperplasia (BPH), who had a transurethral resection of the prostate (TUR-P) and reported histological negative results; or (3) individuals without PCa diagnosis and with PSA levels less than 4 ng/mL and no family history of prostate cancer.

Subjects were excluded from this study if they had a known allergy to seafood, malabsorption, impaired coagulation, or other severe internal diseases, psychiatric or disorders of the CNS, and subjects who were already taking supplementary n-3 FAs.

### 2.3. Treatment Protocol

MPL was obtained from Membramed Health Food GmbH (trade name: Vitalipin). It is a salmon roe extract consisting of 29% phosphatidylcholine and approximately 70% neutral lipids. The n-3 FA profile is 18% eicosapentaenoic acid (EPA) and 26% docosahexaenoic acid (DHA) bound to PLs and neutral lipids [19]. It is formulated in soft gelatine capsules (500 mg/capsule), providing 223 mg EPA and 256 mg DHA daily (1/3 as PLs and 2/3 as TGs).

Participating subjects were asked to take one 500 mg capsule MPL (Vitalipin) three times a day with their meals for a period of 3 months. Each subject received a patient diary in which capsule intake and fish consumption were to be documented weekly. Patients were interviewed before and after MPL intervention. During each interview subjects were surveyed for their nutritional habits (including their fish consumption), medical family history, current medication, and/or intake of supplements. Blood samples were collected for blood routine analysis, lipid electrophoresis, and plasma FA analysis.

### 2.4. Blood Sampling and Analysis

Blood samples were collected from each included subjects before and after MPL supplementation for determining blood parameters, FA analysis, and LPC analysis.

Blood parameters were determined after collecting EDTA blood samples (1 × S-Monovette 2.7 mL with 1.6 mg EDTA/mL blood, Sarstedt, Nümbrecht, Germany) and 1 serum tube (1 × S-Monovette 9 mL, Sarstedt, Nümbrecht, Germany) before and after MPL supplementation. The following parameters were determined: prostate specific antigen (PSA), C-reactive protein (CRP), aspartate transaminase (AST), alanine transaminase (ALT), cholinesterase (CHE), albumin, leukocytes, thrombocytes, triglycerides, total cholesterol, very low density lipoproteins (VLDL), low density lipoproteins (LDL), and high density lipoproteins (HDL), which were determined in the clinical chemistry routine laboratory according to standard procedures.

For FA analysis blood samples were collected in EDTA tubes (1 × S-Monovette 9 mL with 1.6 mg EDTA/mL blood, Sarstedt, Nümbrecht, Germany) and centrifuged for 10 min at 2000 rpm at room temperature, the resulting plasma was stored in aliquots of 500 *μ*L at −80°C until analysis.

After lipid extraction of blood plasma with the methods of Bligh and Dyer (extraction with chloroform/methanol), [20] the total lipid fraction was separated into two fractions with solid-phase extraction (SPE, over an aminopropyl column), namely, the phospholipids and the neutral lipids, according to the procedure validated and described by Taylor et al. [21]. Afterwards, FA analysis was performed with gas chromatography (GC) according to Taylor et al. [21]. GC analysis required derivatization; therefore all fractions were previously methylated with TMSH (Macherey & Nagel, Düren, Germany). Analysis was performed with a HP 5890 Series II Plus, equipped with an Agilent Technologies (Böblingen, Germany) DB-23 column (30 m, 0.25 mm ID, 0.25 *μ*m) with helium at 1 mL/min, oven temperature programming starting with 150°C for 3 min, up to 220°C with a rate of 5°C/min, 220°C for 3.5 min, split injection (1 : 100), injector temperature 260°C, and FID at 280°C.

LPC analysis was performed with high performance thin layer chromatography (HPTLC) after lipid extraction (as mentioned above) from blood plasma according to the method described by Taylor et al. [17]. The plasma samples and five calibration standard solutions of LPC (ranging from 80 to 400 *μ*M, Sigma, Steinheim, Germany) were dissolved in NaCl aqueous solution and extracted with chloroform/methanol. Dry extracts (plasma and calibration standard solutions) were dissolved in hexane/isopropanol/H_2_O and applied to a preconditioned HPTLC plate (Merck, Darmstadt, Germany) with the Camag Automatic Sampler TLC III. After development, plates were dried and stained with a copper sulfate/phosphoric acid solution. Quantification was performed with a Camag TLC-Scanner II equipped with a tungsten bulb at 530 nm.

### 2.5. Statistics

The statistical analysis was performed with SigmaStat 3.10 (Systat Software Inc., USA, 2004) and SPSS 15.0 for Windows. Distribution was analysed with the Shapiro-Wilk test. Student's *t*-test and one-way analysis of variance (ANOVA) were performed if data were normally distributed; otherwise Mann-Whitney *U* test and Kruskal-Wallis one-way ANOVA on ranks were used.

## 3. Results

**Figure 1:**
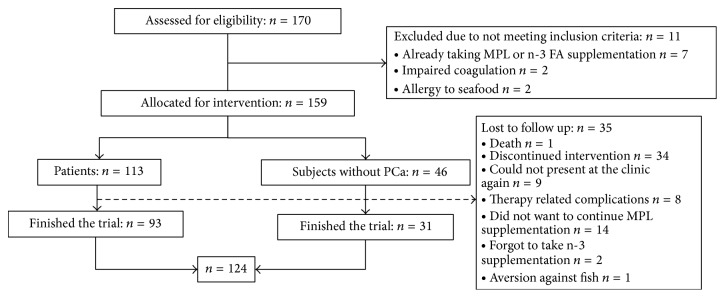
Flow chart of the Prostagen study.

The recruitment totalled 159 subjects, out of which 124 finished the study (Figure 1 and Table 1).

The results presented hereafter are based on a per-protocol analysis (*n* = 124). Most PCa patients had a transurethral resection of the prostate (TUR-P) or a radical retropubic prostatectomy (RRP) and were in remission after surgery. Subjects without PCa had mainly benign prostate hyperplasia (BPH) and were recruited also after surgical intervention (mainly TUR-P, which confirmed the absence of PCa). Since the control subjects had to be at least 70 years old to comply with the inclusion criteria, they had a slightly higher median age than patients.

**Figure 2:**
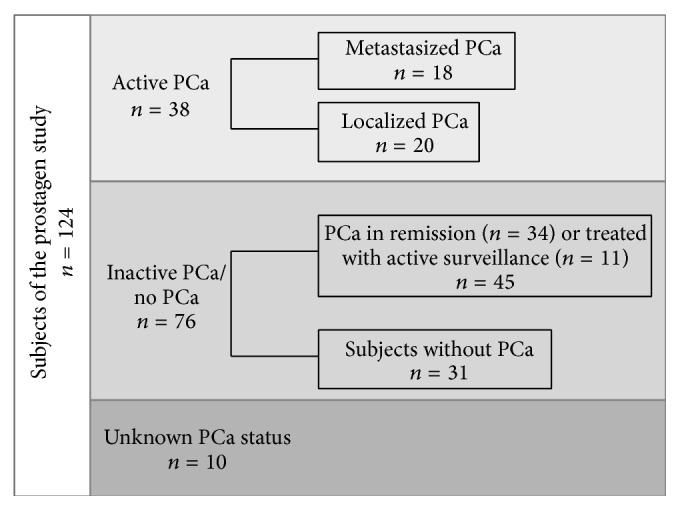
Study population grouped according to PCa diagnosis.

For the analysis of results, subjects were grouped according to their diagnosis taken from their medical records (Figure 2). The first group included patients with active PCa (*n* = 38), which were further divided into patients with metastasized PCa (*n* = 18) and localized PCa (*n* = 20). In both, metastasized and localized PCa, the tumour was growing and patients were subjected to tumour therapy. The second group of subjects was defined by patients with inactive or no PCa (*n* = 76), including patients with PCa in remission—in most cases after surgery—and patients under active surveillance or without therapy in the past 6 months (*n* = 45), and subjects without PCa (*n* = 31). The third group included subjects for whom the medical condition could not be clearly defined (*n* = 10, most of this patients did not agree to undergo further diagnosis) and were therefore not considered when analysing differences between the groups of subjects; otherwise, all subjects were included into the analysis.

### 3.1. MPL Effects on Blood Routine Analysis

Blood routine analysis shows significant changes in most parameters after MPL supplementation. It is important to mention that PSA values are to be evaluated with reservation, since most patients underwent surgery during the intervention. Besides the fact that many factors influence PSA values, the significant decrease observed during this study (Table 2) is probably attributed to the tumour therapy and not to MPL supplementation. Also, since most subjects had surgery a few days before study enrolment, their inflammatory markers like CRP were elevated before MPL supplementation. Its normalization (decrease) observed after the intervention should not be attributed to MPL supplementation but rather to the usual decline after surgical intervention.

### 3.2. Analysis of Nutritional Habits

Each subject's nutritional habits were determined before and after MPL intervention, including their regular consumption of meat, butter, fish, cheese, eggs, and vegetable oil (Quantities of each food product were grouped in >2/week, 1-2/week, 1-2/month, or no intake at all. Only the following fish products (due to their high n-3 FA content) were considered: salmon, herring, tuna, and mackerel.). The reported nutritional habits confirmed the assumption of the low fish intake in the south-western region of Germany. According to other studies analysing fish consumption (e.g., Augustsson et al. [2]), we defined a high fish intake as ≥3 portions of fish per week. The present study population had an average of 1 portion of fish per week. The intake of food products was analysed in relation to the lipid and fatty acid level, without finding any significant associations.

### 3.3. Compliance with MPL Supplementation

The compliance with MPL supplementation was good. Ten subjects (8%) reported fish-oil belching from time to time, which caused no discomfort, and only 11 subjects (9%) occasionally had fish-oil taste after MPL ingestion. Other effects which might be related to MPL supplementation were fish-oil smell in urine and/or stool (2 subjects, 1.6%), bloating and stomach discomfort (2 subjects, 1.6%), reduced coagulation (Coagulation parameters were not measured during the study. Two subjects reported slightly more bleeding after injury and/or easy hematoma formation.) (2 subjects, 1.6%), general fish-oil smell (1 subject, 0.8%), and improved male potency (1 subject, 0.8%). There were no complications resulting in noncompliance of MPL supplementation.

### 3.4. Influence of MPL on Blood Lipids

Blood lipids were measured at baseline and after 3 months of MPL supplementation. It was not possible to draw blood samples in a fasting state since subjects were mostly recruited after having breakfast. Since triglycerides and VLDL are sensible to food intake, the values are not shown.

The study population had normal median cholesterol levels before MPL supplementation (Cholesterol reference values according to the DGFF (Lipid Liga) e.V.). Differences on the initial values of cholesterol were analysed in the groups of subjects revealing no significant differences. Subjects taking lipid lowering medication (about 38% of subjects) had significantly lower LDL initial values than all other participants, whereas HDL remained unaffected.

After MPL supplementation, the measured total cholesterol, LDL, and HDL levels increased significantly in the whole study population about 15% (Table 3). The change of LDL and HDL levels was not affected if subjects were prescribed lipid lowering medication.

**Figure 3:**
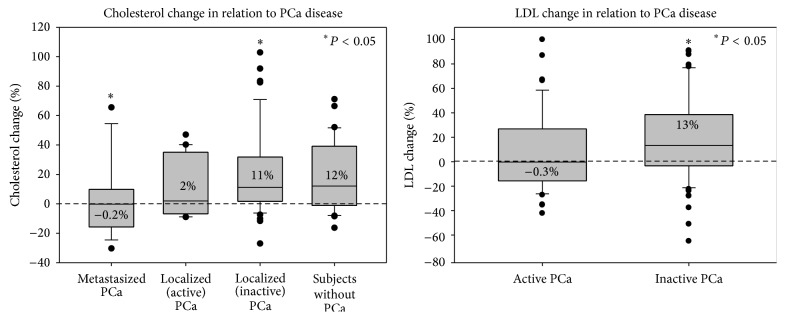
Total cholesterol and LDL change in the different groups of subjects. Left figure: Median relative change of total cholesterol after 3 months of MPL supplementation in each group of subjects. The results show a statistically significant difference (*P* < 0.05) between patients with metastasized PCa (*n* = 18) and patients with PCa in remission/active surveillance (*n* = 45). Right figure: Median relative change of LDL cholesterol after 3 months of MPL supplementation in subjects with active PCa (*n* = 38) and inactive PCa (*n* = 76). There is a statistically significant difference (*P* < 0.05) between both groups of subjects.

Differences in the lipid change between each group of subjects (Figure 2) were analysed. The results show that patients with metastasized PCa had almost no change in their total cholesterol levels after MPL supplementation, whereas all other patients and subjects without PCa had a significant increase. Detailed analysis showed that these differences were found mainly in the LDL fraction of total cholesterol (Figure 3). In regard to HDL cholesterol change, no significant differences were found between each group of subjects.

### 3.5. Influence of MPL on the Fatty Acid (FA) Composition in Plasma

Nine FAs in each subject before and after MPL supplementation were analysed (Table 4). Since the plasma sample of one patient could not be used for the FA analysis, the results were based only on a study population of 123 subjects.

**Figure 4:**
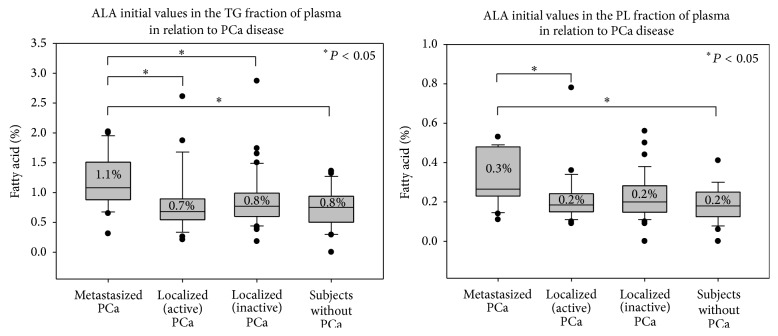
Initial values of ALA in the TG and PL fractionsbetween the different groups of subjects. Median relative initial values of ALA in the TG and PL fractions of blood plasma. Significance was tested between each group of subjects. Significant differences (*P* < 0.05) were observed between the highlighted (∗) groups of subjects.

Initial values of FAs were analysed between each group of subjects (mentioned in Figure 2). As seen in Figure 4, initial values of *α*-linolenic acid (ALA) were significantly higher in patients with metastasized PCa in contrast to all other subjects in both the TG and PL fractions.

DHA values in the TG fraction of plasma were also significantly different between the groups of subjects. Patients with metastasized PCa had a median relative value of 0.9%, whereas subjects without PCa had 0.5%. No significant differences were observed for the initial values of all other FAs between the groups of subjects.

Table 4 shows the proportion of plasma FAs before and after MPL supplementation and their respective relative change. Almost all FA proportions changed significantly with MPL supplementation.

**Figure 5:**
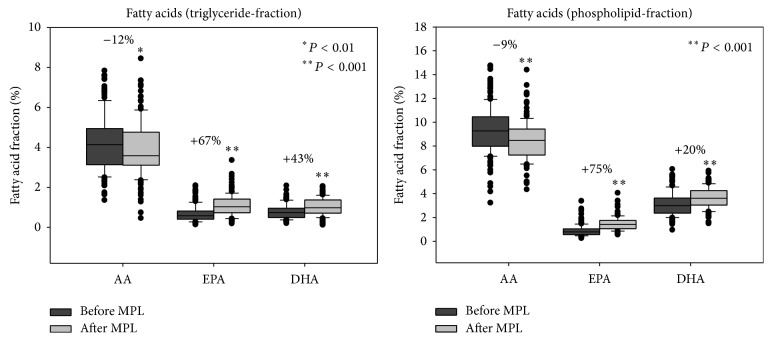
Long chain n-3 and n-6 FAs before and after MPL supplementation in the TG and PL fractions of plasma. Median relative values of the biologically active FAs AA, EPA, and DHA in the TG and PL fractions of plasma before and after 3 months of MPL supplementation in the study population (*n* = 123). The AA (n-6) decreased significantly after supplementation, whereas EPA and DHA increased significantly in both fractions. Although AA in the TG fraction and DHA in the PL fraction had a normal distribution all FAs are represented in median values for comparison.

From the biologically active n-3 and n-6 FAs, EPA and DHA increased significantly after MPL supplementation in both the TG and PL fractions of plasma, whereas the AA decreased significantly. Therefore, the n-3/n-6 FA ratio improved significantly from 0.38 to 0.58 ((EPA + DHA)/AA in the TG and PL fractions, with *P* < 0.01) (Figure 5).

Linoleic acid (LA, 18 : 2, n-6 FA) and ALA (18 : 3, n-3 FA), which are precursors of the biologically FAs, EPA, and AA, were shown to increase significantly (*P* < 0.01) in both the TG and the PL fractions of plasma, after MPL supplementation.

Differences on the FA change between each group of subjects (mentioned in Figure 2) were analysed, but no significant differences were found.

### 3.6. Influence of MPL on Lysophosphatidylcholine (LPC) in Plasma

Initial values of LPC were not significantly different between the groups of subjects and had a median value of 181 *μ*M. After MPL supplementation LPC increased in the whole study population to 223 *μ*M (*P* < 0.001). When observing the LPC change in the different groups of subjects, patients with metastasized PCa had a LPC decrease after MPL supplementation, while in all other groups LPC increased. The difference between the group of patients with metastasized PCa and all others was significant (*P* < 0.05).

## 4. Discussion

During this clinical trial we found that simulating a high fish consumption in subjects with a usually rather low fish consumption by supplying MPL improved the n-3/n-6 FA ratio significantly. This was observed in all study groups without differences. MPL supplementation showed to have a good compliance compared to fish oil supplementation described in other clinical trials. For example, the study of Bruera et al., in which fish oil was supplemented to patients in an advanced cancer stage, showed to cause multiple gastrointestinal problems with an even lower dose [22].

Besides the MPL-induced increase of the n-3/n-6 FA ratio in all study groups, significant differences on the LDL—and lysophosphatidylcholine (LPC)—increase between the groups of subjects were observed. Patients with metastasized PCa/active PCa did not show the LDL and LPC accumulation, which was present in all other groups, probably due to a higher demand of the metastasizing tumour cells for these lipids. Another significant difference between both groups of subjects was found in the initial values of ALA, possibly due to a higher eicosanoid biosynthesis from n-6 FA.

To our knowledge until today, there is no evidence showing that actively growing PCa cells have a higher LDL uptake, but there are clues that LDL plays a role in the development of PCa. An* in vitro* study showed that the growth inhibiting effect of simvastatin on prostate cancer cell lines was prevented when adding LDL [23]. The mechanisms by which LDL could be contributing to PCa development remain unclear, but it might be possible that LDL receptors are overexpressed in tumour cells, facilitating its uptake to comply with their lipid demand for proliferation. For example, Gilardoni et al. observed in rats that the expression of LDL-Receptor-related Protein-1 (LRP-1), which is one LDL receptor type, was higher in premalignant lesions compared to normal cells [24]. In general, a higher cholesterol demand by tumour cells has been linked to cancer progression through, for example, increased cellular proliferation, inflammation as well as regulating lipid rafts and thereby affecting signalling pathways of apoptosis. In consequence, high circulating cholesterol levels were associated with an increased risk of aggressive PCa [25]. Hence, our results are in line with studies that showed reduced cholesterol levels in men developing cancer [26–28], since tumour cells could be using cholesterol for their survival.

The probable higher LPC uptake by metastatic tumour cells observed during our study corresponds with* in vitro* experiments showing that metastatic tumour cells rapidly catabolize LPC as well as incorporate it into their cellular membranes, thereby changing its membrane FA composition [29]. Also, Raynor showed that PCa cell lines (PC3, DU145, and LNCaP) had a higher LPC incorporation and metabolism than normal cells [30]. During clinical trials, it was found that LPC levels were decreased in patients with an advanced cancer disease [17] and in patients with colorectal cancer [31]. Thus, the probable high LPC demand of aggressively growing tumour cells would explain the missing LPC increase observed in the group of patients with metastasized PCa. However, it would have been consistent if LPC values before giving MPL were also lower in metastasized PCa patients when compared to all other subjects, but no differences were observed before intervention. An explanation could be that all subjects had already low LPC values (181 *μ*M, normal values range from 200 to 400 *μ*M [32–35]).

It has been shown that eicosanoid synthesis from PUFAs is increased in tumour cells [36] and that the relative proportion of n-3 and n-6 PUFAs in the cellular membranes is one factor which determines the types of eicosanoids that are generated [5]. The n-6 FA AA has been described to be implicated in the development of cancer and also of metastases through the synthesis of series 2 eicosanoids, especially of PGE_2_ [5, 6, 37]. With the results of this study we speculate that during metastasis an increased conversion of AA to eicosanoids takes place, since the values of *α*-linolenic acid (ALA) before MPL supplementation were higher in patients with metastasized PCa compared to all other subjects. This finding supports the assumption that metastasized/active tumour cells “consume” more n-6 FAs than n-3 FAs, resulting in accumulation of the n-3-FA ALA, which is the precursor of the n-3 FA EPA.

In concordance with this finding, lower initial values of the n-6 FA linoleic acid (LA, precursor of AA) would have been expected in patients with metastasized PCa as a consequence of a higher AA demand. While in other studies reduced proportions of LA have been found in cancer patients in comparison to healthy individuals [21] in this study no differences could be observed between the groups of patients. One explanation might be that due to the usually high AA intake in the western diet of all study participants, an increased conversion of LA to AA might not be required to fulfil the needs of the metastatic tumour cells. In addition, LA blood concentration is much higher than that of ALA (LA/ALA in PL is approximately 80, in TG approximately 34) and, thus, a possible small reduction of LA in relation to its high values cannot be clearly observed.

Based on our results, a hypothesis is proposed (Figure 7) which helps to explain the prevention of metastatic tumour growth in more than 40% of PCa patients that have high fish consumption, which was shown in different clinical trials [2, 3].

**Figure 6:**
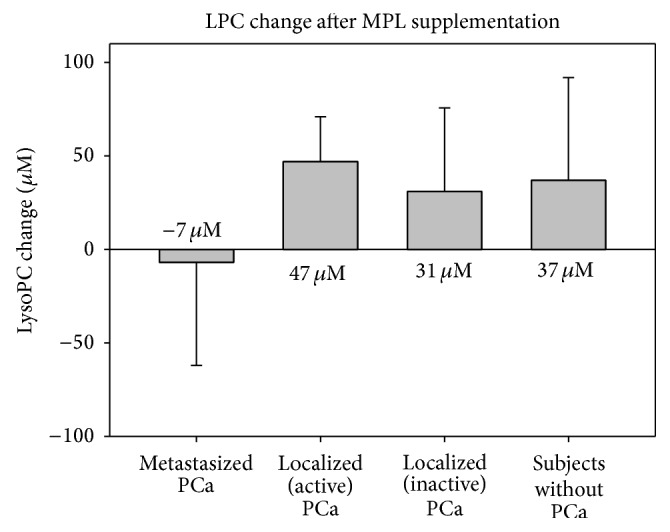
LPC change in the different groups of subjects.

**Figure 7:**
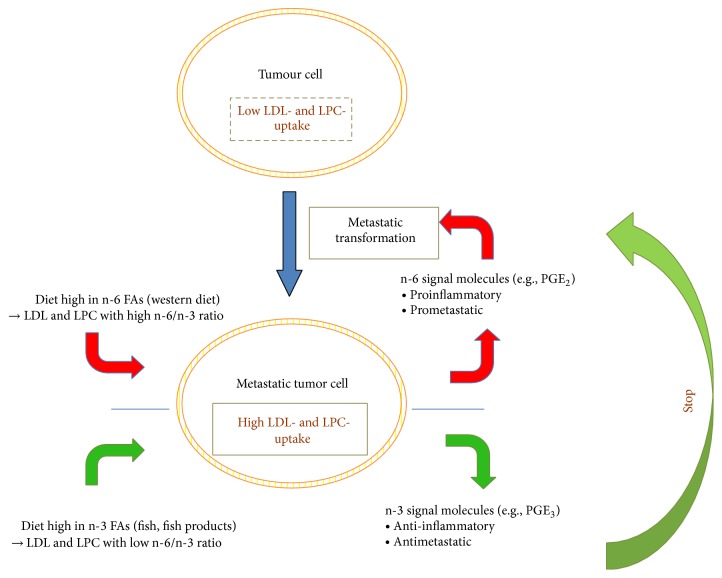
Hypothetic model explaining the “*omega-sensitivity*” found in about half of the patients with metastatic PCa.

From our data we assume that aggressively growing PCa cells developed the ability to take up high amounts of LDL and that during metastatic transformation those cells developed the additional property to take up and metabolize high amounts of LPC (Figure 6). Thus, LDL and LPC are expected to especially supply metastatic cancer cells with high amounts of FAs.

Depending on which kind of FAs are predominantly contained in the diet (“western diet:” rich in n-6 FAs versus fish diet (or MPL): rich in n-3 FAs), the FA composition of LDL and LPC will be modulated accordingly. Therefore, we assume that the diet influences the n-3/n-6 FA ratio especially in metastatic tumour cells, influencing thereby whether cells synthesize either pro- or antimetastatic eicosanoids. LDL and LPC could be regarded as* Trojan horses*, which become only dangerous to the metastasizing tumour cells if they were previously enriched with n-3 FAs. Patients, who respond to either western diet or fish diet with an increase in LDL and LPC, containing the respective pro- or antimetastatic FA, are therefore considered as “omega-sensitive.”

## 5. Conclusions

Taken together MPL supplementation was shown to be effective in increasing the n-3/n-6 FA ratio significantly, with a very good compliance at the same time. With our results we could assume that active/metastatic tumour cells have an influence on the LDL and LPC levels, probably due to a higher demand of these lipids. Influencing the FA composition of LDL and LPC towards a higher n-3/n-6 FA ratio could convert LDL and LPC particles into* Trojan horses*, having the potential to influence the n-3/n-6 FA ratio of tumour cells and thereby reduce the biosynthesis of proinflammatory eicosanoids. If the results of this clinical trial could be confirmed with further investigations, a high fish intake is recommended to PCa patients, since it was shown that 40–50% of patients profit from this diet to impede metastatic development [3]. If achieving high fish consumption is not possible, MPL showed to be an efficient alternative in delivering n-3 FAs. These results warrant further studies to confirm the effects of an n-3 FA increase in relation to PCa development.

## Figures and Tables

**Table 1:** Baseline characteristics of the study population.

Study population *n* = 124			
Patients		Subjects without PCa	
*n* = 93		*n* = 31	
Age (years)	68 (48–83)	Age (years)	74 (70–85)^a^
median (min–max)		median (min–max)	
BMI (kg/m^2^)	26 (20.6–41.6)	BMI (kg/m^2^)	26.3 (22.2–34)^b^
median (min–max)		median (min–max)	
PCa disease (*n*)		Urological condition (*n*)	
Metastasized PCa	18	BPH	21
PCa	20	Kidney stones	2
PCa in remission	45	PCa biopsies with neg. result	2
Unknown PCa status	10	High grade PIN^h^	2
		Other^i^	4
Gleason score (*n*)			
≤7a	56		
≥7b	58		
Therapy (*n*)^c^		Therapy (*n*)^j^	
Chemotherapy^d^	6	Surgery^f^	22
Radiotherapy^e^	2	No therapy	9
Hormone therapy	15		
Surgery^f^	53		
Active surveillance	7		
No therapy^g^	10		

^a^Age difference between both groups was statistically not significant (*P* = 0.099).

^b^BMI difference between both groups was statistically not significant (*P* = 0.46).

^c^Referred to the ongoing therapy at study enrolment.

^d^Docetaxel, taxol, folfox, or taxotere.

^e^Including brachytherapy.

^f^TUR-P or RRP with or without lymphadenectomy.

^g^No therapy in the last 6 months.

^h^High-grade prostatic intraepithelial neoplasia.

^i^Strangury, renal hematoma, bladder neck stenosis, and hematuria.

^j^TUR-P, transurethral incision of the prostate (TUIP), percutaneous nephrolithotomy, bladder neck incision, spermatocelectomy, open adenoma enucleation, transurethral laser vaporization, or placement of prostatic stent.

**Table 2:** Blood parameters in the study population before and after MPL supplementation.

Blood parameter	Before MPL	After MPL∗	*P*
Albumin (g/dL)	4.1 (2.8–5)	4.3 (2.6–5)	<0.001
Erythrocytes (Mio/*μ*L)	4.2 (2.7–5.7)	4.6 (2.8–5.7)	<0.001
Haematocrit (%)	38.7 (22.7–50.3)	41.9 (24.7–50.3)	<0.001
CRP (mg/L)	12 (0.7–260)	2 (0.7–305)	<0.001
PSA (*μ*g/L)	2.3 (0–1085)	0.4 (0–1117)	<0.001
Thrombocytes (1000/*μ*L)	219 (27–481)	218 (99–484)	n.s.
Leucocytes (1000/*μ*L)	7.2 (3.2–16.4)	6.3 (2.8–24.1)	<0.001
CHE (U/L)	7419 (2185–13950)	8221 (3959–12635)	<0.001
AST (U/L)	28.5 (9–316)	28 (16–79)	n.s.
ALT (U/L)	26 (5–170)	26 (6–120)	n.s.

∗Most patients underwent surgery shortly before MPL intervention.

**Table 3:** Blood lipids before and after MPL supplementation.

	Before MPL (mg/dL)	After MPL (mg/dL)	Change (%)
Total cholesterol median (min–max)	194 (80–349)	221 (115–362)	+14%∗∗
LDL cholesterol median (min–max)	121 (36–277)	139 (42–282)	+15%∗∗
HDL cholesterol median (min–max)	49 (23–106)	57 (21–125)	+16%∗∗
LDL : HDL mean ± SD	2.8 ± 1.3	2.7 ± 1.4	—

Analysis performed with lipid electrophoresis with nonfasting blood samples. Mean ± SD values are shown for normally distributed data, otherwise median (min–max) values were given.

∗∗Significance level *P* < 0.001.

**Table 4:** Results of fatty acid analysis with GC.

Fatty acid	Before MPL (%)	After MPL (%)	Relative change of respective FA^†^	*P*
TG-fraction				
Myristic acid-C14:0	1.9 (0.5–5.6)	2.2 (0.7–23.5)	+16%	<0.01
Palmitic acid-C16:0	23.9 (14.7–35.7)	23.2 (14.7–36.9)	−3%	<0.05
Stearic acid-C18:0	3.7 (1.5–8)	3.7 (1.9–7.2)	0%	n.s.
Oleic acid-C18:1	34.1 (25.4–43.2)	32 (23.1–45.3)	−6%	<0.01
Linoleic acid (LA)-C18:2	27.6 (12.9–46.1)	29.6 (15.8–47.9)	+7%	<0.01
*α*-Linolenic acid (ALA)-C18:3	0.8 (0.1–9.6)	1 (0–5)	+25%	<0.001
Arachidonic acid (AA)∗-C20:4	4.1 (1.4–7.8)	3.6 (0.5–8.4)	−12%	<0.01
Eicosapentaenoic acid (EPA)-C20:5	0.6 (0.1–2.1)	1 (0.2–3.4)	+67%	<0.001
Docosahexaenoic acid (DHA)-C22:6	0.7 (0.2–2.1)	1 (0.1–2.1)	+43%	<0.001
∑ of median values	97.4	97.3		

PL-fraction				
Myristic acid-C14:0	0.3 (0.1–0.7)	0.4 (0.2–0.7)	+33%	<0.001
Palmitic acid-C16:0	25.4 (15.9–30)	24.9 (20.5–28.6)	−2%	<0.01
Stearic acid-C18:0	11.7 (8.4–16.3)	12.1 (8.4–15.8)	+3%	<0.001
Oleic acid-C18:1	10.6 (5.6–14.2)	10.7 (7.4–15.6)	0%	n.s.
Linoleic acid (LA)-C18:2	17.4 (11.2–24.3)	18.5 (8.3–23.9)	+6%	<0.01
*α*-linolenic acid (ALA)-C18:3	0.2 (0–3.4)	0.3 (0–0.9)	+50%	<0.001
Arachidonic acid (AA)∗-C20:4	9.3 (3.2–14.8)	8.5 (4.3–14.4)	−9%	<0.001
Eicosapentaenoic acid (EPA)-C20:5	0.8 (0.2–3.4)	1.4 (0.5–4.1)	+75%	<0.001
Docosahexaenoic acid (DHA)-C22:6	3 (0.9–6.1)	3.6 (1.5–5.9)	+20%	<0.001
∑ sum of median values	78.7	75.4		

∗Normal distribution.

^†^Change of FAs after MPL supplementation based on the median initial values.

Median relative values of each FA before and after MPL supplementation in the study population (*n* = 123). Since most of the data is not normally distributed, all values are given in median (min–max). Since C:17 lysophosphatidylcholine was used as internal standard, the results of C:17 are not shown in the table. C17 stays mostly in the PL fraction; therefore the values of the shown FAs (% of each FA) are not based on a 100%.
